# Korean Mistletoe (*Viscum album* var. *coloratum*) Ethanol Extracts Enhance Intestinal Barrier Function and Alleviate Inflammation

**DOI:** 10.3390/antiox14030370

**Published:** 2025-03-20

**Authors:** Ye Jin Yang, Min Jung Kim, Ji Woong Heo, Hun Hwan Kim, Gon Sup Kim, Min Sub Shim, Kwang Youn Kim, Kwang Il Park

**Affiliations:** 1 College of Veterinary Medicine, Gyeongsang National University, 501 Jinjudaero, Gazwa, Jinju 52828, Republic of Korea; yang93810@gnu.ac.kr (Y.J.Y.); minjung0102@gnu.ac.kr (M.J.K.); hujiw7806@gnu.ac.kr (J.W.H.); hunskim@gnu.ac.kr (H.H.K.); gonskim@gnu.ac.kr (G.S.K.); 2 Department of Biochemistry and Molecular Genetics, College of Graduate Studies, Midwestern University, 19555 N. 59th Ave., Glendale, AZ 85308, USA; mshim@midwestern.edu; 3 Korean Medicine (KM)-Application Center, Korea Institute of Oriental Medicine, 70 Cheomdanro, Dong-gu, Daegu 41062, Republic of Korea

**Keywords:** Korean mistletoe (*Viscum album* var. *coloratum*), intestinal barrier function, tight junction, ulcerative colitis, inflammatory bowel disease

## Abstract

Korean mistletoe (*Viscum album* var. *coloratum*, KML) offers remarkable therapeutic potential for a variety of diseases. This study aims to evaluate the effects and potential molecular mechanisms of KML ethanol extracts (KMLE), focusing on intestinal barrier function and tight junctions (TJs) in an interleukin (IL)-6-induced Caco-2 cell monolayer model and a dextran sodium sulfate (DSS)-induced ulcerative colitis (UC) mouse model. KMLE is non-cytotoxic in Caco-2 cells and demonstrated strong antioxidant activity. KMLE alleviated significant barrier dysfunction and protected tight junction proteins (TJPs) in vitro. Furthermore, KMLE alleviated clinical symptoms and histopathological damage, upregulated TJPs, and suppressed the inflammatory cytokines in vivo. Additionally, six bioactive compounds were identified in KMLE by liquid chromatography–tandem mass spectrometry (LC-MS/MS). In conclusion, KMLE ameliorated intestinal barrier dysfunction in vitro and in vivo. These findings underscore the potential of KMLE as a therapeutic agent for UC, providing insights into the mechanisms through anti-inflammatory properties and its ability to restore TJ integrity.

## 1. Introduction

IBD (inflammatory bowel disease) refers to a set of intestinal disorders, the most common of which are ulcerative colitis (UC) and Crohn’s disease (CD) [1]. IBD first appeared in newly developed countries such as Asia, South America, and the Middle East and has since spread to every continent with increasing incidence [2,3]. Symptoms associated with IBD include diarrhea, rectal bleeding, stomach pain, and weight loss [4,5], which can thwart career goals, foster social stigma, and lower patients’ quality of life [2]. IBD is caused by intestinal barrier failure that mostly impacts the surface layers of the colon, and the large intestine experiences continuous chronic inflammation that disrupts the intestinal lamina’s barrier function and may result in systemic infection [6]. Most IBD patients present with reduced and broken tight junction (TJ) chains [7].

Also, the barrier function of the intestinal epithelium is maintained partially by TJs between adjacent epithelial cells [8]. TJs between enterocytes protect the intestinal epithelial barrier and play a crucial role in cell permeability [9]. TJs function as paracellular gates, which are built by a network of protein interactions in the lateral membrane’s apical region [10]. TJs consist of TJ proteins (TJPs), such as zonula occludens (ZO), filamentous actin, occludins, claudins, junctional adhesion molecules (JAMs), and other transmembrane proteins, and are dynamically complex with multiple functions [8,11,12]. The abnormal expression of TJPs such as ZO-1, occludin (OCLN), and claudin-1 in intestinal tissue promoted intestinal permeability and pathogen infiltration and induced immunologic dysfunction and the development of IBD [13,14]. Therefore, the protection of TJs is important to maintaining intestinal homeostasis [14].

The usual medical treatments for TJs for colitis are surgery and medications. The most common clinical treatment medications are 5-aminosalicylic acid (5-ASA), immunosuppressants, biologics, and corticosteroids [15]. 5-ASA compounds, such as sulfasalazine (SSZ), olsalazine (OSZ), and mesalamine (MSM), are anti-inflammatory pharmaceuticals used to treat UC [15,16]. However, long-term use of these steroid medications can result in a variety of adverse effects, including liver and renal damage, drug resistance, and allergic responses, as well as a progressive loss of pharmacological effectiveness depending on the individual [17]. Therefore, because of these problems with steroid-based treatments, there is increasing interest in using traditional herbs to treat ulcerative colitis. Traditional herbs have beneficial effects and fewer side effects.

Korean mistletoe (KML) is a plant in the mistletoe family Loranthaceae that is native to Korea. KML is chemically composed of viscotoxins, lectins, flavonoids, phenolic acids, terpenoids, sterols, phenylpropanoids, and alkaloids [18]. KML promotes antioxidant effects and increases endurance through increased mitochondrial activity [19]. In addition, KML is often used in traditional medicines for various purposes. Recently, it has been reported to be effective not only for its anti-cancer [20], anti-tumor [21], and antioxidant effects [22,23]; it has also been recognized for its immune enhancement [24], immune-regulatory [25], and anti-inflammatory effects [26] with respect to the human gut system. However, research on the TJ to mistletoe is still lacking for KML.

Many studies have proven that inflammatory cytokines such interleukin (IL)-1β, IL-6, tumor necrosis factor (TNF)-α, and interferon (IFN)-γ have a negative effect, weakening the intestinal barrier function, and they are employed in models of cellular intestinal barrier failure [15,19,27]. This study aimed to determine whether KML extracts (KMLE) play a role in maintaining the integrity of the intestinal epithelial barrier. Given the critical function of this barrier in preventing IBD, elucidating the protective effects of KMLE may provide critical implications for therapeutic interventions. Specifically, this study sought to investigate the influence of KMLE on intestinal barrier function and to elucidate its underlying mechanisms, with a particular emphasis on the regulation and activation of tight junctions.

## 2. Materials and Methods

### 2.1. Reagents

Cell culture reagents and consumables were acquired from GIBCO (Grand Island, NY, USA). Fluorescein isothiocyanate-dextran 4kDa (FITC-dextran 4) was from Thermo scientific (Waltham, MA, USA). IL-6 protein was from NKMAX (Seongnam, Geonggi, Republic of Korea). The Cell Counting Kit (CCK)-8 was from NKMAX (Seongnam, Geonggi, Republic of Korea). The RNA extraction kit was from Qiagen (Hilden, Germany). An ELISA kit was purchased from R&D systems (Minneapolis, MN, USA). Rabbit polyclonal anti-ZO-1 and mouse polyclonal anti-OCLN were from Invitrogen (Thermo scientific, Waltham, MA, USA). Mouse monoclonal anti-β-actin was from NKMAX (Seongnam, Geonggi, Republic of Korea).

### 2.2. Sample Preparations

KML was purchased from the herbal market in Daegu, the Republic of Korea. Dried samples were ground for extraction. The KML extraction process involved extraction with ethanol for 24 h and freeze-drying for 1 day. The freeze-dried Korean mistletoe extracts (KMLE) were stored at 4 °C, and the stock of 100 mg/mL made with distilled water (DW) was stored at −20 °C. KML ethanol extraction was named KMLE. The stock solution of standards was prepared in DW at a concentration of 1 mg/mL of KMLE, which was accurately weighed and subsequently diluted in DW. All standard solutions containing KMLE were stored at either 4 °C or −20 °C. To prepare for high-performance liquid chromatography (HPLC) analysis, all working solutions were filtered through a 0.45 μm syringe membrane filter (GVS, Sanford, ME, USA).

### 2.3. Cell Culture and Treatment

The Caco-2 human colon cancer cell line was acquired from the Korean Cell Line Bank (KCLB, Seoul, Republic of Korea). The cells were cultured in high-glucose minimum essential medium (MEM), supplemented with 10% fetal bovine serum (FBS) and 1% penicillin, and incubated at 37 °C in a 5% CO_2_ atmosphere. The culture medium was replenished every 1 to 2 days to maintain optimal growth conditions. Trypsinized cells were added to 100π cell culture-treated dishes (9000 μL; SPL, Gyeonggi, Republic of Korea), to 60π cell culture-treated dishes (3000 μL; SPL, Gyeonggi, Republic of Korea), to 24-well cell culture-treated plates (1000 μL; SPL, Gyeonggi, Republic of Korea), to 96-well cell culture-treated plates (100 μL; SPL, Gyeonggi, Republic of Korea), or to insert-well filters (6.5 mm × 0.33 cm^2^, 0.3 μm pore size; SPL, Gyeonggi, Republic of Korea).

### 2.4. Measurement of Antioxidant Efficacy

To evaluate the free radical scavenging ability of KMLE, the DPPH and ABTS assays were performed with modifications [28,29]. DPPH is employed as a reagent to provide a simple and precise method for titrating the oxidizable groups of natural or synthetic antioxidants. Ascorbic acid (AA) was used as a standard antioxidant (positive control). A volume of 190 μL of 0.2 mM DPPH methanolic solution was added to each well in a 96-well plate, followed by the addition of 10 μL of the sample, 100 μg/mL AA, or a solvent for blank controls. The mixture was incubated at 37 °C for 30 min, and absorbance was measured at 517 nm using a microplate reader (BioTek, Winooski, VT, USA). ABTS radicals were generated through an oxidation reaction involving potassium persulfate. To ensure freshness, radical stock solutions were prepared immediately before use. The reaction was carried out in the dark at 20 °C for 5 min. To achieve an absorbance of 0.70 ± 0.02 at 734 nm, the concentration of the blue-green ABTS radical solution was adjusted using methanol.

### 2.5. Cell Viability Assay

Cell viability was assessed using the CCK-8 assay in accordance with the manufacturer’s guidelines. Caco-2 cells (1 × 10^4^ cells) were seeded into a 96-well plate. Following treatment with IL-6 or its absence, along with KMLE, CCK-8 solution (10 μL) was added to each well, and the reaction was incubated under standard culture conditions for 1 h. The absorbance at 450 nm was subsequently measured using a Cytation7 (BioTek, Winooski, VT, USA).

### 2.6. Measurement of Transepithelial Electrical Resistance (TEER)

Caco-2 cells were plated at a density of 2 × 10^4^ cells in culture medium in 0.33 cm^2^ polyethylene terephthalate membrane insert wells and incubated for a duration of 21 days. The culture medium was replaced every 2 days until full differentiation was achieved. Electrical resistance was assessed using the Millicell-ERS electrical resistance system EVOM3 (World Precision Instruments, Sarasota, FL, USA). The epithelium’s apical and basolateral sides were washed by 1× phosphate buffered saline (PBS), pH 7.4, for the measurement. Caco-2 cells were treated or not treated with 100 and 200 μg/mL KMLE 1h prior to the addition of 50 ng/mL IL-6, and the TEER was measured at every 6 h for 24 h. TEER values were expressed as Ohm (Ω) cm^2^.

### 2.7. Epithelial Paracellular Permeability

Paracellular permeability was assessed using the nonabsorbable FITC-dextran 4. Following the TEER experimental treatments, both the apical and basolateral compartments were rinsed with PBS. Subsequently, FITC-dextran 4 (1 mg/mL) was added to the apical compartment, and PBS was added in the basolateral compartment. After incubating at 37 °C for 1 h, basolateral medium was transferred to a 96-well plate and measured using the Cytation7 (BioTek, Winooski, VT, USA). Excitation and emission wavelengths were determined to be 490 nm and 520 nm, respectively.

### 2.8. Immunofluorescence (IF) Staining

Cells were exposed to 100 and 200 μg/mL KMLE prior to 1h IL-6 50 ng/mL. After the experimental treatment, Caco-2 cells were rinsed twice in PBS then fixed by 4% formaldehyde in PBS for 10 min and permeabilized with 0.25% Triton X-100 in PBS for 10 min at 20 °C. Cell monolayers were incubated in 5% bovine serum albumin (BSA), dissolved in PBS for 1 h at 20 °C to prevent non-specific binding, and incubated overnight at 4 °C with primary antibodies rabbit polyclonal anti-ZO-1 (1:100, Invitrogen, Waltham, MA, USA) and mouse polyclonal anti-OCLN (1:100, Invitrogen, Waltham, MA, USA), followed by secondary antibody incubation with FSD^TM^ 488 and FSD^TM^ 594 (1:100, BioActs, Incheon, Republic of Korea) and DAPI counterstaining. After that, TJPs were visualized, and images were obtained using Cytation 7 (BioTek, Winooski, VT, USA).

### 2.9. RNA Extration and Real-Time PCR Analysis

The 1 × 10^6^ Caco-2 cells/well were seeded in 60π dishes. At the end of the experiment, Caco-2 monolayers were promptly rinsed with iced-PBS, and RNA extraction using an RNA extraction kit (Qiagen, Hilden, Germany) was undertaken. An amount of 1 μg of total RNA was reverse transcribed to cDNA using the QuantiTect Revers Transcription Kit (Qiagen, Hilden, Germany). Real-time quantitative PCR was conducted using the AccuPower^®^ 2X GreenStar qPCR master mix (Bioneer, Daejeon, Republic of Korea) under the following conditions: initial denaturation at 95 °C (10 min), followed by 40 cycles of denaturation at 95 °C (15 s), annealing at 60 °C (1 min), and extension at 65 °C (5 s), with a final extension period at 95 °C (1 min) using a CFX qPCR (Biorad Laboratories, Hercules, CA, USA). Quantification of mRNA levels for each sample was performed by analyzing the cycle threshold (Ct) values. Target gene expression levels were normalized to the reference gene, glyceraldehyde 3-phosphate dehydrogenase (GAPDH). The sequences of primers is as follows: TNF-α (NM_000594.4; Forward: 5′-ACATACTGACCCACGGCTTC-3′; Reverse: 5′-GCACTCACCTCTTCCCTCTG-3′), C-C motif chemokine ligand 5 (CCL5, NM_002985.3; Forward: 5′-TGCTGCTTTGCCTACATTG-3′; Reverse: 5′-CACTTGGCGGTTCTTTCG-3′), C-C motif chemokine ligand 17 (CCL17, NM_002987.4; Forward: 5′-CTGATGAGCCTCAGGTGACA-3′; Reverse: 5′-CCAGGATGCTCTCAGTCACA-3′), and GAPDH (NM_002046.7; Forward: 5′-GAAGGTGAAGGTCGGAGT-3′; Reverse: 5′-CATGGGTGGAATCATATTGGAA-3′).

### 2.10. Animals

The C57BL/6J mice, 5 weeks old (Male, 20–22 g), were purchased from Samtako Inc. (Osan, Geonggi, Republic of Korea). The mice were housed under 23 ± 3 °C and relative humidity condition (56 ± 4%) with a 12h/12h light and dark cycle. Standard diet (Orientbio Inc., Sungnam, Geonggi, Republic of Korea) and drinking water were provided ad libitum to the mice. All experimental animal procedures were approved by the Korea Institute of Oriental Medicine Institutional Animal Care and Use Committee (KIOM-D-19-007) and were conducted according to the guidelines of the National Institutes of Health (NIH publication).

### 2.11. Animal Experiments

**Figure 4:**
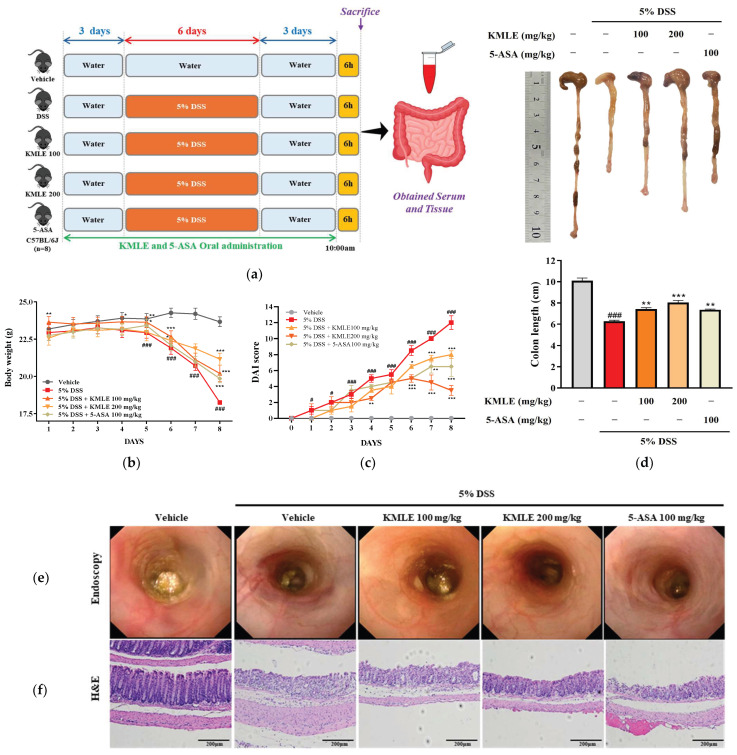
Clinical symptom relief effect and histological analysis of KMLE. (**a**) Animal experiment schedule. The experiments were performed for 11 days. The normal water was provided for 3 days before 5% DSS administration for days. The 5% DSS-containing normal water was provided to mice for 5 days. After 5 days, it was changed to normal water for 3 days. And 100 and 200mg/kg of KMLE and 100mg/kg 5-ASA were orally administered for 11days. On the last day of the experiments, tissue and blood were obtained from mice. (**b**) Changes in body weight (*n* = 8) and (**c**) the disease activity index (DAI) score following KMLE and 5-ASA administration from DSS-induced experimental period in colitis mice (*n* = 8). (**d**) Colon length shortening during DSS-induced and KMLE (100 or 200 µg/mL) or 5-ASA (100 mg/kg) administration during the experimental periods (*n* = 8). (**e**) Image of DSS-induced mucosal damage using an endoscope (*n* = 8). (**f**) Confirmed H&E-stained colon section images shown at 200× magnification (*n* = 4). Scale bar = 200 μm. Statistical analysis was performed according to one-way or two-way ANOVA with Dunnett’s multiple comparisons test. Data are expressed as the mean ± SEM. # *p <* 0.05, ### *p* < 0.001 versus the vehicle group; * *p* < 0.05, ** *p* < 0.01, *** *p* < 0.001 versus the DSS-induced group.

A total of 40 mice were randomly assigned to five groups: the vehicle group, the 5% dextran sodium sulfate (DSS; MW 36,000–50,000, MP Biomedicals, Montreal, QC, Canada) group, the KMLE 100 mg/kg + 5% DSS group, the KMLE 200 mg/kg + 5% DSS group, and the 5-ASA 100 mg/kg + 5% DSS group (positive control). The animal experiment was conducted over 12 days. KMLE (100 mg/kg or 200 mg/kg) and 5-ASA (100 mg/kg) were administered orally to the respective groups once daily for 12 consecutive days. All groups received normal drinking water for the first three days. Colitis was induced by administering 5% DSS in drinking water for six days in all groups except the vehicle group. Following DSS treatment, mice were provided with normal drinking water for an additional three days. The vehicle group received normal drinking water throughout the entire experimental period. Body weight was measured daily before oral gavage of KMLE and 5-ASA. At the end of the experiment, following a six-hour fasting period, blood samples were collected under isoflurane anesthesia (isoflurane, Hana Pharm. Co., Ltd, Gyeonggi, Republic of Korea), and serum was isolated. Subsequently, colon tissues were harvested via cervical dislocation (Figure 4a).

### 2.12. Clinincal Symptoms and DAI Scoring

The Disease Activity Index (DAI) was evaluated at consistent time points following the initiation of 5% DSS administration, starting from day 0. The assessment was based on three clinical parameters: body weight loss, stool consistency, and rectal bleeding. The DAI was calculated as the sum of the individual scores for each parameter, according to the following formula: DAI = weight loss score + stool consistency score + bleeding score. Each category was scored on a scale reflecting severity, with higher scores indicating more severe symptoms (Table 1). Specifically, body weight loss was scored as follows: no weight loss (0); 1–5% loss (1); 5–10% loss (2); 10–15% loss (3); 15–20% loss (4); and >20% loss (5). Stool consistency was graded as normal (0); loose stools (1–2); diarrhea (3); or absence of stools (4). Rectal bleeding was evaluated as none (0); slightly bloody with light or dark brown coloration (1); moderate bleeding with dark brown stools (2); significant bleeding with red diarrhea (3); and gross hemorrhaging with bright red blood throughout the colon (4).

### 2.13. Endoscopy and Hematoxylin and Eosin (H&E) Staining

To evaluate the histological changes in the intestinal epithelial barrier of DSS-induced mice, images were acquired using a veterinary endoscopy system (120 mm length and 2.0 mm diameter mini-endoscope, SPiTZ VET CARE, Tuttlingen, German) on mice under anesthesia. Following the endoscopic procedure, the collected intestinal tissues were fixed in a 4% paraformaldehyde solution, embedded in paraffin blocks, and sectioned using a microtome. The prepared histological sections were then stained with H&E for analysis.

### 2.14. Quantification of Protein Levels

The 1 × 10⁶ Caco-2 cells/well were seeded in 60π dishes and rinsed with PBS at the end of the experiment. Cells and tissues were lysed using radioimmunoprecipitation assay (RIPA) buffer (Thermo Scientific Fisher, Waltham, MA, USA) containing a 1× protease inhibitor cocktail (GenDEPOT, Katy, TX, USA). Cell lysates were centrifuged at 12,000 rpm for 15 min at 4 °C, and the supernatant was collected for protein quantification using the BCA protein assay kit (BIOMAX, Gyeonggi, Republic of Korea). For SDS-PAGE, a 5× sample buffer (ELPis, Daejeon, Republic of Korea) was added to equal amounts of protein and heated at 80 °C for 13 min. Protein samples (20 μg each) were separated on a 10% gel, transferred to a polyvinylidene fluoride (PVDF) membrane (Millipore Corporation, Billerica, MA, USA), and blocked in 5% skimmed milk in TBS containing 0.05% Tween-20 for 2 h. The membrane was incubated overnight at 4 °C with primary antibodies (anti-ZO-1, anti-OCLN, anti-β-actin, and anti-GAPDH, each at 1:1000). After washing, the membrane was incubated with secondary antibodies for 2 h at room temperature. Protein bands were visualized using an enhanced chemiluminescence detection system (Thermo Fisher Scientific, Waltham, MA, USA) and analyzed using the Bio-Image Analysis System (Image J, version 1.53t, National Institutes of Health, Bethesda, MD, USA).

### 2.15. Enzyme-Linked Immunosorbent Assay (ELISA)

As directed by the manufacturer’s instructions, cytokine concentrations were determined using the corresponding ELISA kits (R&D Systems, Minneapolis, MN, USA). For cell experiments, the cell supernatant was absorbed and centrifuged at 3000 rpm at 4 °C for 20 min before use. To generate the standard curve, multiple concentrations (50 μL each) were added to the respective standard wells. Each sample well added 10 μL of material, which was then diluted to 40 μL. All wells (except for the blank well) received 100 μL of horseradish peroxidase (HRP)-labeled antibody. After sealing the reaction holes with sealing plate film, these were incubated for 1 h at 37 °C. After this, the solution was discarded and each well was washed with a washing solution. A total of 50 μL of substrate A and 50 μL of substrate B were added to each well and incubated at 37 °C for 15 min, shading the light. A total of 50 μL of stop solution was added to each well and, 15 min later, measured at 450 nm.

### 2.16. Analysis of Liquid Chromatography with Tandem Mass Spectrometry (LC-MS/MS)

LC-MS/MS analysis of KMLE was carried out using a Nexera ultra-performance liquid chromatography (UPLC) system (Shimadzu, Kyoto, Japan) coupled with an X500R quadrupole time-of-flight mass spectrometer (QTOF MS, AB SCIEX, Framingham, MA, USA), employing a Pronto SIL 120-5-C18 SH column (150 × 4.6 mm, 5 μm; Bischoff Chromatography, Leonberg, Germany). The solvent used was distilled water (solution D) and 0.1% formic acid (Supelco, Bellefonte, PA, USA) in acetonitrile (Sigma Aldrich, St. Louis, MO, USA) (solution ACN). The solvent conditions used in the mobile phases were as follows: 0–10 min at 10–15% solution ACN; 10–20 min at 20% solution ACN; 20–30 min at 25% solution ACN; 30–40 min at 40% solution ACN; 40–50 min at 70% solution ACN; 50–60 min at 95% solution ACN; and 60–70 min at 95% solution ACN. The analysis was performed using electrospray ionization (ESI)–mass spectrometry under the following conditions: detection wavelength of 284 nm; column temperature of 35 °C; flow rate of 0.5 mL/min; and injection volume of 3 μL. Samples were analyzed in negative ion mode, and data acquisition was carried out using SCIEX OS software version 3.0 (SCIEX, Framingham, MA, USA).

### 2.17. Molecular Docking Analysis for KMLE

Molecular docking is a robust approach for assessing receptor–ligand interactions, particularly those involving enzymes associated with tight junctions (TJ) of compounds. This method was employed to investigate the potential binding modes of the ZO-1 ligand and the inhibitory effects of chlorogenic acid, quinic acid, caffeic acid, syringin, quercetin 3-vicianoside, and dimethoxyluteolin on these interactions. Protein structures, experimentally determined and available in the Research Collaboratory for Structural Bioinformatics (RCSB) Protein Data Bank (PDB), were retrieved for the docking analysis (https://www.rcsb.org/, accessed on 23 September 2024). Specifically, the unliganded Zona Occludens protein 1 (ZO-1, ID: 4OEO) PDZ1 domain was used. The 3D structures of chlorogenic acid (CID: 1794427), quinic acid (CID: 6508), caffeic acid (CID: 689043), syringin (CID: 5280507), quercetin 3-vicianoside (CID: 44259139), and dimethoxyluteolin (CID: 5351234) were obtained from PubChem (https://pubchem.ncbi.nlm.nih.gov/, accessed on 23 September 2024). The docking simulations were performed using the AutoDock Vina module (version 1.1.2, default settings) and UCSF Chimera 1.17.3. The results were visualized with Discovery Studio 4.0 software, and the binding affinities were calculated based on total intermolecular energy and estimated free energy of binding. All experiments were conducted in triplicate, with a root-mean-square deviation (RMSD) of ≤2 Å.

### 2.18. Statistical Analysis

All statistical analyses were conducted using GraphPad Prism 8.0 (GraphPad Software, San Diego, CA, USA). Group comparisons were conducted using one-way or two-way factorial analysis of variance (ANOVA) to determine statistical differences. Data are presented as the mean ± standard error of the mean (SEM). Statistical significance was defined as a *p*-value < 0.05.

## 3. Results

### 3.1. KMLE Has Non Cytotoxicity and Antioxidants Effect

**Figure 1:**
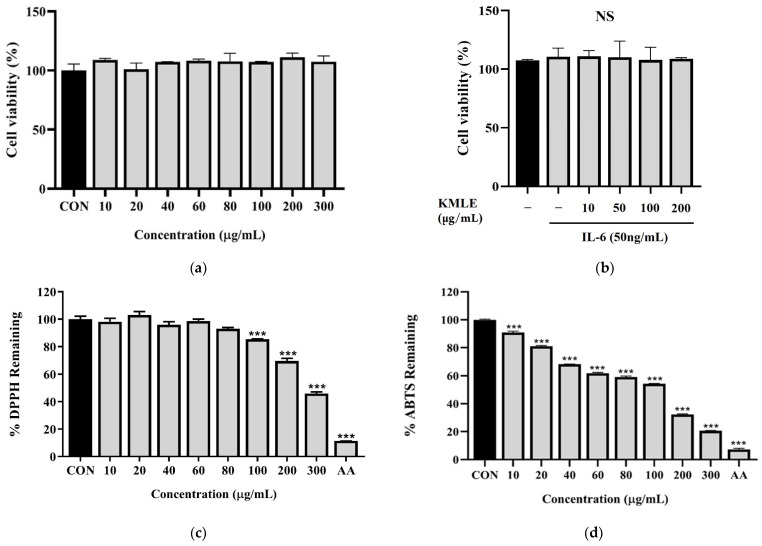
Cell viability of KMLE in Caco-2 cells and antioxidants effect of KMLE. (**a**) KMLE was used at various concentrations for 24 h. (**b**) Pretreatment for 1 h with IL-6 (50 ng/mL) and treated KMLE (10, 50, 100, and 200 μg/mL) and incubation for 24 h. (**c**) The DPPH free radical scavenging activity of the KMLE was measured with DPPH methanolic solution. (**d**) The ABTS radical scavenging activity of the KMLE was measured with ABTS solution. CON: control; AA: ascorbic acid 100 µg/mL. All data are derived from at least three independent experiments and are presented as representative results. Statistical analysis was performed according to one-way ANOVA with Dunnett’s multiple comparisons test. Data are expressed as the mean ± SEM. Not significant (NS); *** *p* < 0.001 versus CON group.

To evaluate the effect of KMLE in Caco-2 cells, cell viability was evaluated at various concentrations of KMLE via the CCK-8 assay after incubating the cells for 24 h in the presence or absence of IL-6 (50 ng/mL). The results showed that KMLE concentration ranging from 0 to 300 µg/mL, with or without IL-6, was not cytotoxic to Caco-2 cells (Figure 1a,b). Therefore, these two doses were considered safe and used for the further experiments. As a result of measuring the antioxidant efficacy of KMLE through the DPPH procedure and ABTS procedure, significant antioxidant effects were identified. In particular, DPPH showed a significant decrease from 100 to 300 µg/mL of KMLE (Figure 1c). ABTS was shown to have good antioxidant effects from 10 µg/mL of KMLE (Figure 1d).

### 3.2. KMLE Reduced Inflammatory Mediators

**Figure 2:**
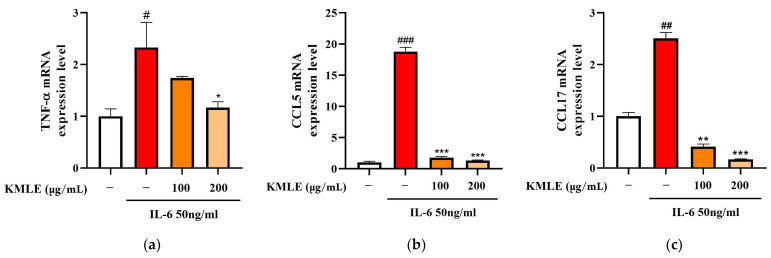
Effect of KMLE on inflammatory medicators mRNA level. (**a**) TNF-α mRNA expression level, (**b**) CCL5 mRNA expression level, and (**c**) CCL17 mRNA expression level detected via real-time PCR analysis. All data are derived from at least three independent experiments and are presented as representative results. Statistical analyses were performed using one-way ANOVA followed by Dunnett’s multiple comparisons test. Data are expressed as the mean ± SEM (*n* = 3). # *p* < 0.05, ## *p* < 0.01, ### *p* < 0.001 versus the control group; * *p* < 0.05, ** *p* < 0.01, *** *p* < 0.001 versus the IL-6 group.

To confirm whether KMLE induced changes in the inflammatory mediators, Caco-2 cells were stimulated with 50 ng/mL IL-6 for 24 h, and the expression levels of inflammatory cytokines and chemokines were measured using qPCR. As the result, TNF-α, CCL, and CCL17 levels were increased in the IL-6 group (Figure 2a–c). On the other hand, 100 and 200 µg/mL of KMLE treatment reduced the TNF-α, CCL5, and CCL17 levels when compared to the IL-6 group (Figure 2a–c).

### 3.3. Effects of KMLE on IL-6-Induced Epithelial Barrier Dysfunction

**Figure 3:**
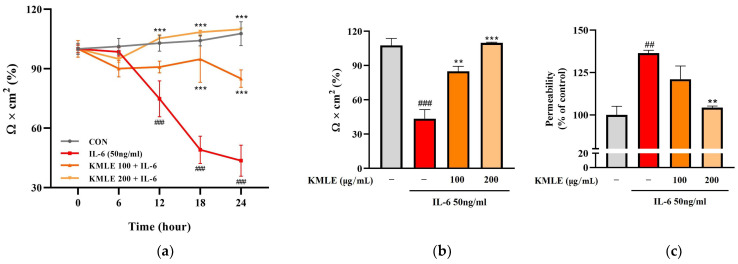

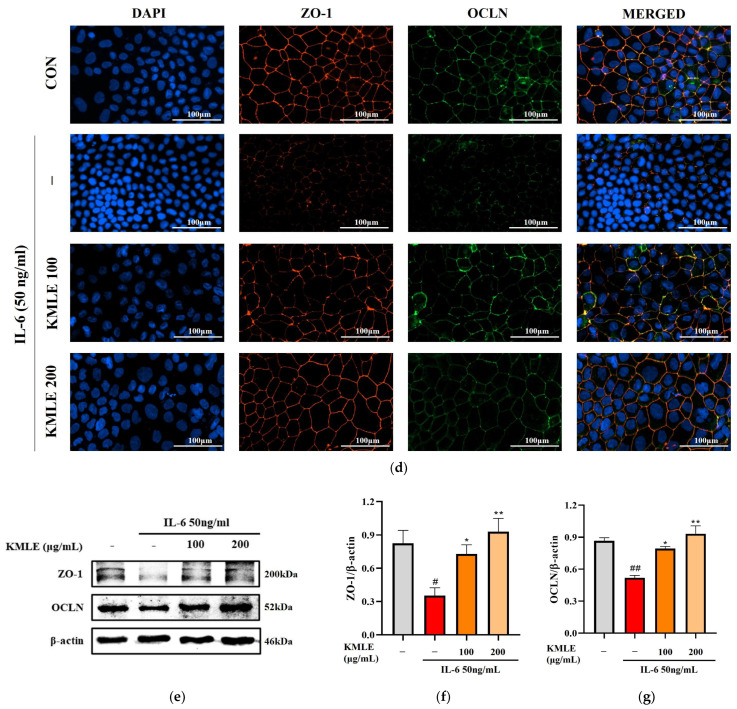
Effects of preventing intestinal barrier function and TJ of KMLE. (**a**) The expression and regulation of epithelial barrier function by KMLE. TEER values of KMLE-treated Caco-2 monolayers. (**b**) The percentage TEER value at 24 h. (**c**) Permeability to FITC-dextran 4 of KMLE-treated Caco-2 monolayers. (**d**) Expression of TJPs by KMLE in the cellular boundary. Immunofluorescence staining for ZO-1 (red) and OCLN (green) protein in Caco-2 monolayer. Scale bar = 100 μm. (**e**) Representative Western blots of TJPs. (**f**) ZO-1 expression level was normalized by β-actin. (**g**) OCLN expression level was normalized by β-actin. All data are derived from at least three independent experiments and are presented as representative results. Statistical analyses were performed using one-way or two-way ANOVA followed by Dunnett’s multiple comparisons test. Data are expressed as the mean ± SEM (*n* = 5). # *p* < 0.05, ## *p* < 0.01, ### *p* < 0.001 versus the control group; * *p* < 0.05, ** *p* < 0.01, *** *p* < 0.001 versus the IL-6 group.

In the IL-6 group, the TEER values tended to steadily decrease over 24 h and decreased rapidly at 18 h (49.063%) compared to 12 h (74.790%) (Figure 3a). After 24 h, these values decreased to 43.577% compared to the control (107.671%) (Figure 3b). However, KMLE protected against the decrease induced by induced IL-6. The decrease in TEER value induced by IL-6 was inhibited in a manner dependent on the KMLE concentration (Figure 3a). In the KMLE 100 μg/mL group, the value was 84.962% at 24 h, lower than the control (102.33%). In KMLE 200 μg/mL, it showed a tendency to increase over time, and the TEER value at 24 h (109.864%) was similar to the control (107.671%) (Figure 3a,b). Additionally, the permeability was confirmed to be reversed with the TEER value (Figure 3b,c). The IL-6 group increased to 136.445% and decreased to 104.312% in KMLE 200 μg/mL (Figure 3c). These results mean that KMLE prevented the impairment of barrier dysfunction by IL-6.

### 3.4. KMLE Prevents IL-6-Induced Morphological Disruption of TJPs in the Epithelial Barrier

The increased secretion of pro-inflammatory mediators may lead to enhanced paracellular permeability by disrupting TJPs, such as ZO-1 and OCLN. To assess the integrity of intercellular junctional complexes, an IF assay was conducted. Under normal conditions, ZO-1 and OCLN were localized at the cell membrane, forming a continuous and uninterrupted boundary around the cells (Figure 3d). However, IL-6 exposure resulted in a disrupted and discontinuous expression pattern of ZO-1 and OCLN at the cell borders. Notably, KMLE treatment restored the continuous expression of these TJPs in a concentration-dependent manner. In particular, 200 μg/mL KMLE exhibited a well-defined TJP expression pattern at the cell boundaries, similar to that observed in the control group (Figure 3d). To further confirm the effect of KMLE on TJs, which are critical for maintaining intestinal barrier integrity, Western blot analysis was performed (Figure 3e). The results indicate that ZO-1 and OCLN expression levels were significantly reduced in the IL-6-treated group compared to the control. However, pretreatment with KMLE effectively increased the expression of ZO-1 and OCLN in a concentration-dependent manner (Figure 3f,g). These findings mean that KMLE mitigates IL-6-induced down-regulation of TJPs in Caco-2 cells and prevents TJ disruption.

### 3.5. KMLE Ameliorates the Clinical Symptoms and Protected Against Destruction of Intestinal Barrier Tissue

We examined the therapeutic effect of orally administered KMLE on the acuteness of DSS-induced colitis. DSS-induced mice had significantly reduced body weight compared with the vehicle group (Figure 4b). However, KMLE administration obviously improved the body weight loss as compared with the DSS-induced group (Figure 4b). Furthermore, consistent with body weight results, DAI score and colon length was restored by KMLE administration compared with that of the DSS-induced group (Figure 4c,d). The morphological and histological changes in the large intestine were evaluated after the administration of DSS and KMLE via endoscopy and H&E to determine the degree of colitis and the effect of KMLE. The DSS-induced group exhibited significant morphological and histological alterations, including severe ulceration of the colonic mucosa, indicative of colon tissue damage (Figure 4e,f). However, this damage was ameliorated following the administration of KMLE, as compared to the DSS-induced group. Furthermore, the number of goblet cells was markedly reduced in the DSS-induced group (Figure 4f). Notably, KMLE administration effectively mitigated goblet cell depletion in comparison to the DSS group. These results mean that KMLE may improve against DSS-induced colitis.

### 3.6. KMLE Regulated Intestinal TJs in DSS-Induced Colitis Mice

**Figure 5:**
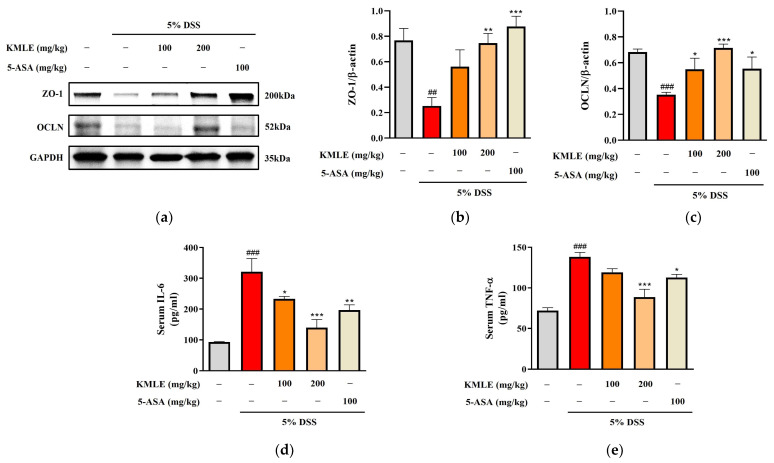
Effects of KMLE on intestinal TJ and down-regulation of inflammatory cytokines. (**a**) ZO-1 and OCLN, TJPs expression by Western blot. (**b**) Expression level of ZO-1 normalized by β-actin. (**c**) Expression level of OCLN normalized by β-actin. Western blot analysis was conducted on colon tissue (*n* = 4). (**d**) The expression level of IL-6 was detected in mice serum. (**e**) The expression level of TNF-α was detected in mice serum (*n* = 8). Statistical analysis was performed according to one-way ANOVA with Dunnett’s multiple comparisons test. Data are expressed as the mean ± SEM. ## *p* < 0.01, ### *p* < 0.001 versus the vehicle group; * *p* < 0.05, ** *p* < 0.01, *** *p* < 0.001 versus the DSS-induced group.

We performed a Western blot analysis to determine whether KMLE regulates the tight junction proteins ZO-1 and OCLN. The ZO-1 expression was reduced in the DSS-induced group, and this was increased by treatment with KMLE (Figure 5a,b). In addition, the protein expression level of OCLN decreased in DSS-induced colitis. However, it was recovered by treatment with KMLE (Figure 5a,c). These results indicated that KMLE prevented the down-regulation of TJPs in the DSS-induced colitis model.

### 3.7. Effects of KMLE on Serum Levels of Pro-Inflammatory Cytokines in DSS-Induced Colitis Mice

Measurements of the cytokine levels of IL-6 and TNF-α were undertaken to evaluated whether KMLE regulates inflammatory cytokines in serum. The levels of both IL-6 and TNF-α were remarkably increased in the DSS-induced group as compared to the control group (Figure 5d,e). However, both levels of IL-6 and TNF-α in the group treated with KMLE were lower than those in the DSS-induced group (Figure 5d,e).

### 3.8. Analysis of LC–MS/MS to Identify Active Compounds in KMLE

**Figure 6:**
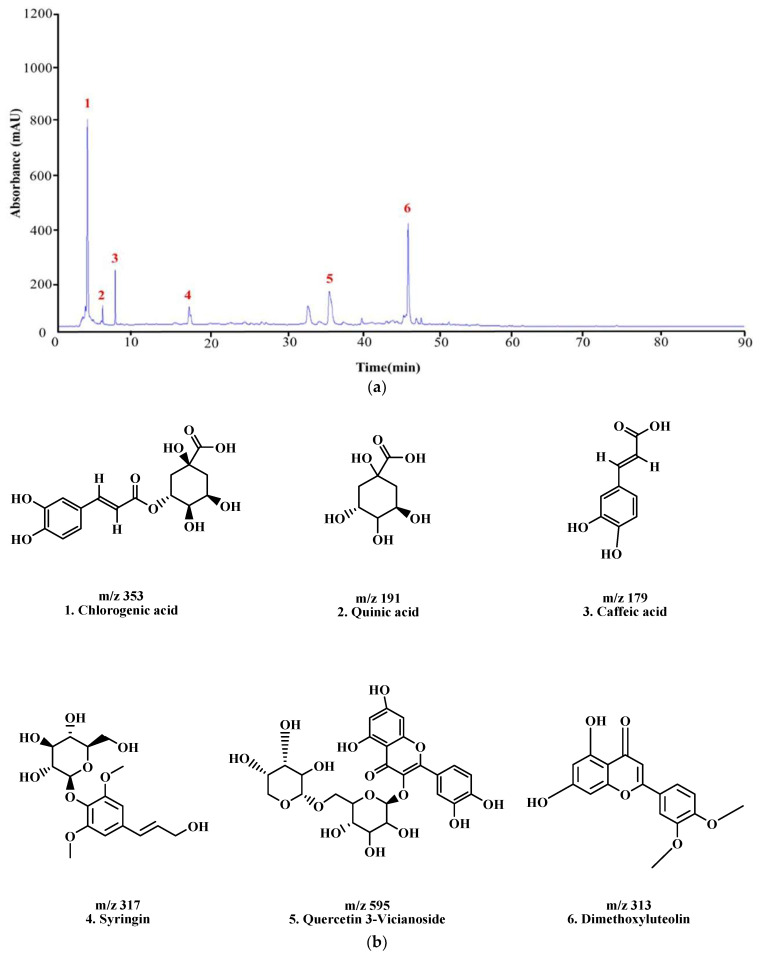

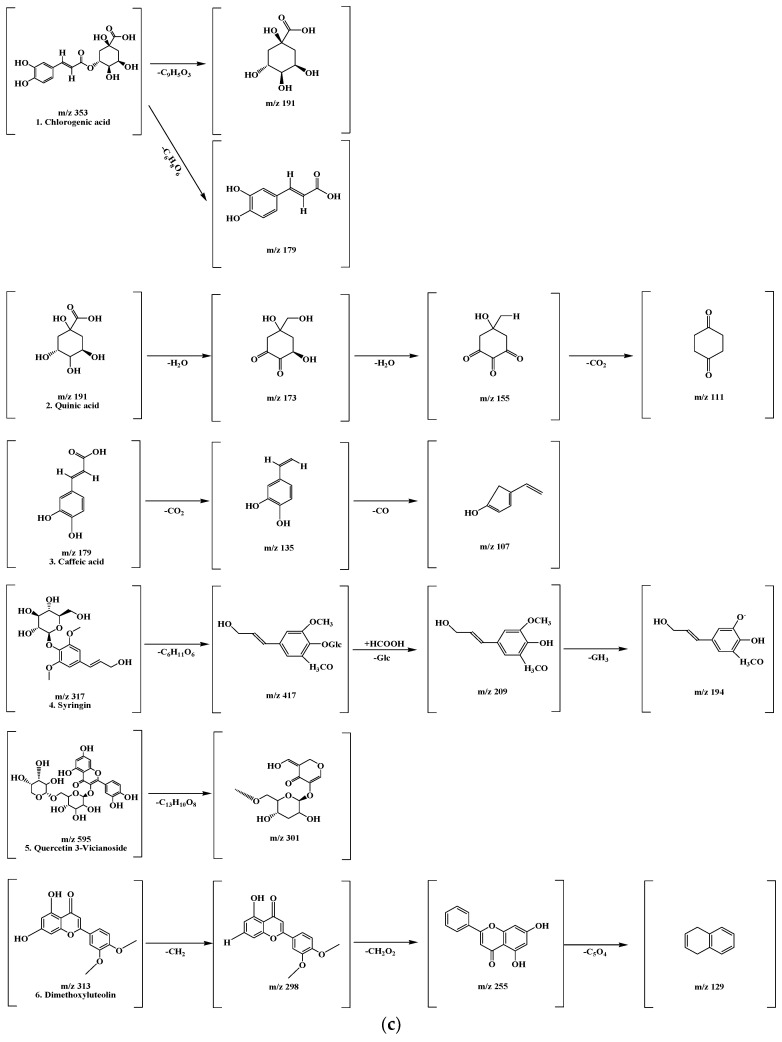
LC-MS/MS chromatogram peaks and chemical structures of six compounds in KMLE. (**a**) LC-MS/MS chromatographic profile of KMLE obtained using gradient elution. The analysis was performed with a sample injection volume of 10 μL and a flow rate of 0.5 mL/min. (**b**) Chemical structures of the six identified compounds in KMLE. (**c**) Proposed fragmentation patterns of chlorogenic acid (*m*/*z* 353), quinic acid (*m*/*z* 191), caffeic acid (*m*/*z* 179), syringin (*m*/*z* 317), quercetin-3-vicianoside (*m*/*z* 595), and dimethoxyluteolin (*m*/*z* 313).

The constituents of KMLE were analyzed through LC-MS/MS analysis: Chlorogenic acid ;1 [30], Quinic acid ;2 [31], Caffeic acid ;3 [32,33], Syringin ;4 [34], Quercetin 3-Vicianoside ;5 [35], and dimethoxyluteolin ;6 [36], respectively, based on the retention times and mass spectra (Figure 6 and Table 2).

### 3.9. Molecular Docking Analysis of KMLE Compounds with ZO-1

**Figure 7:**
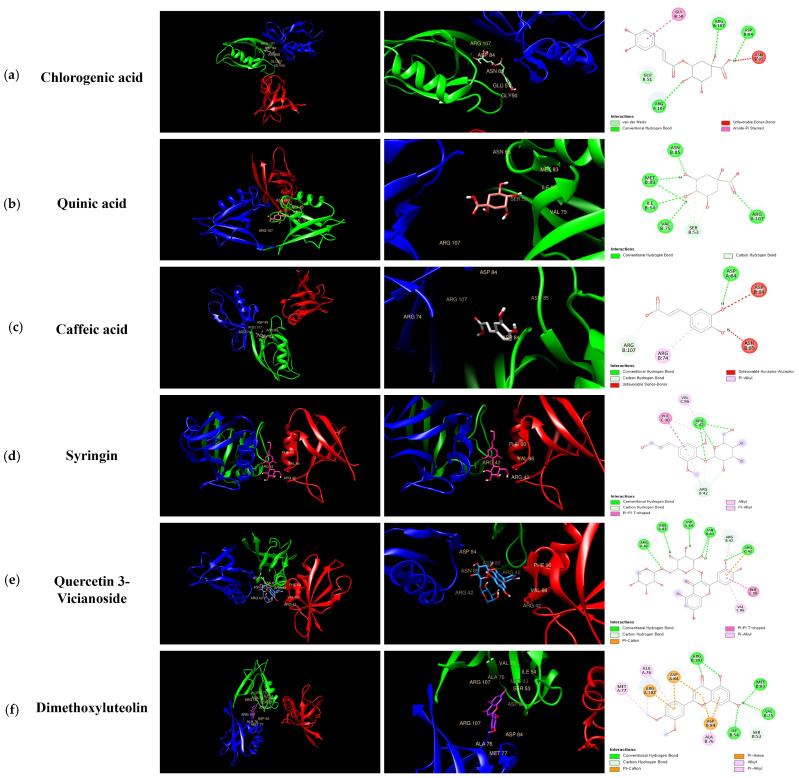
Molecular interactions between KMLE and human TJP ZO-1 (4OEO): (**a**) Chlorogenic acid; (**b**) Quinic acid; (**c**) Caffeic acid; (**d**) Syringin; (**e**) Quercetin 3-vicianoside; (**f**) Dimethoxyluteolin. These were docked onto the 3D structure of ZO-1, with each color corresponding to a specific type of direct interaction.

Molecular docking was performed with ZO-1 using six compounds—Chlorogenic acid (a); Quinic acid (b); Caffeic acid (c); Syringin (d); Quercetin 3-vicianoside (e); and Dimethoxyluteolin (f)—identified in Figure 7. Additionally, it was shown that the docking scores illustrate the binding interactions between each protein and ligand at their complementary surfaces (Figure 7) and quantify the binding energy required for these interactions, as presented in Table 3.

## 4. Discussion

The incidence of UC is related to multiple factors. Damage to the mechanical barrier of the intestinal mucosa is a prerequisite for triggering abnormal intestinal immunity, inflammation, and ulcer formation [37]. It is the initiating factor and core link in the progression of UC, as well as a key target for alleviating UC [38].

The present study was designed to evaluate whether KMLE protects intestinal barrier function and to investigate its regulatory effects on TJPs in a DSS-induced colitis mouse model and Caco-2 cells. Previous studies have demonstrated that cytokines such as IL-6 [39], IL-1β [40], and TNF-α [41] caused intestinal barrier dysfunction in the epithelial cells through inflammation. Among many cytokines, we investigated the effect of KMLE on IL-6-stimulated Caco-2 cells (Figure 1, Figure 2 and Figure 3).

The use of multiple antioxidant assays for the determination of the antioxidant activity of a plant extract can provide a comprehensive understanding of the antioxidant properties of extracts [29]. As the results show, KMLE has an excellent antioxidant effect (Figure 1c,d). In the present study, we demonstrated that KMLE inhibits the reduction in the expression of TJPs, such as ZO-1 and OCLN, in IL-6-stimulated Caco-2 cells (Figure 3d,e). In vivo studies have demonstrated that DSS-induced colitis serves as a representative animal model for intestinal inflammatory diseases, presenting pathophysiological features comparable to those of human disorders [42]. KMLE treatment reduced clinical symptoms such as loss of body weight (Figure 4b), DAI score (Figure 4c), intestinal shortening (Figure 4d), and pathological grade in mice (Figure 4f).

Conventional IBD treatment focuses on symptom management through pharmacotherapy, including aminosalicylates, corticosteroids, immunomodulators, and tumor necrosis factor (TNF) inhibitors [43]. However, while there are patients who have seen the effects of these agents, there remain patients in which they are insufficiently effective or should be discontinued because of adverse side effects [44]. Many recent studies have suggested that herbal medicines possess validated therapeutic potential in protecting intestinal barrier function [45,46]. In this study, we determined the effect of KMLE on intestinal barrier dysfunction compared with 5-ASA. In the present study, we confirmed that DSS induced inflammation in the intestinal barrier and that KMLE reduced this inflammation (Figure 4e). Also, it was confirmed that KMLE recovered the epithelium ultrastructure destruction in a DSS-induced colitis mouse model through histological staining (Figure 4f).

In acute colitis, pro-inflammatory cytokines (IL-6, IL-1β, and TNF-α) and chemokines (CCL5 and CCL17) are released, promoting macrophage and neutrophil recruitment and activation, ultimately leading to lymphocyte proliferation [47,48,49]. Accordingly, we confirmed cytokines’ and chemokines’ mRNA levels in vitro; the KMLE reduced inflammatory cytokine/chemokines’ mRNA levels in Caco-2 cells (Figure 2). Additionally, as the results confirm the presence of IL-6 and TNF-α in the serum of the DSS-induced colitis mouse model, KMLE reduced the cytokines in the serum of the DSS-induced colitis mouse model (Figure 5d,e). The other major finding of this study is the upregulation of TJPs, including ZO-1 and OCLN, following KMLE treatment in vitro and in vivo (Figure 3e and Figure 5a). ZO proteins were among the first identified TJ-specific proteins, and to date, three isoforms have been characterized: ZO-1, ZO-2, and ZO-3 [50,51]. ZO-1 interacts with ZO-2 and ZO-3, while its PDZ domain plays a crucial role in anchoring receptor proteins to the membrane and linking them to intracellular structures [52,53]. TJP ZO-1 is a key protein for the intestinal mucosal mechanical barrier [54,55]. The results show that ZO-1 protein expression was higher in the intact intestinal epithelial barrier than in the damaged one, with significantly increased fusion to the cell membrane (Figure 3a). This result is consistent with the findings of recent studies, which have found that the fusion of ZO-1 with intestinal epithelial cell membranes is a necessary condition for maintaining the integrity of the intestinal mucosal mechanical barrier [11,56]. Furthermore, we evaluated the affinity of KMLE and ZO-1 to regulate TJ via molecular docking. The molecular docking study revealed that six compounds of KMLE formed several hydrogen bonds and hydrophobic interactions with amino acid residues of the ligand-binding cavity of ZO-1; strong binding forces were confirmed in the order of quercetin 3-vicianoside, dimethoxyluteolin, chlorogenic acid, syringin, caffeic acid, and quinic acid (Figure 7 and Table 2). Among them, compounds that showed a binding affinity of -7 or higher were quercetin 3-vicianoside, dimethoxyluteolin, and chlorogenic acid (Table 3). Quercetin 3-vicianoside and dimethoxyluteolin are flavonoids with strong antioxidant effects [57,58], and chlorogenic acid and caffeic acid are polyphenols with antioxidant and anti-inflammatory properties [59,60,61]. Syringin is a glycoside linkage leading to unique biological effects [62]. Quinic acid is known as a precursor to phenolic acid metabolism and antioxidant activity [63]. Therefore, KMLE containing these compounds is suggested to have excellent antioxidant and anti-inflammatory effects.

In summary, the KMLE effect in a DSS-induced colitis mouse model and Caco-2 cells works by suppressing the inflammatory-related chemokine levels and recovering the protein levels of ZO-1 and OCLN. These results suggest the potential of the KMLE to improve intestinal barrier function and inhibit inflammation in IBD. Therefore, the KMLE might be useful as a new therapeutic agent for preventing colitis through its anti-inflammatory properties, and it could ameliorate dysfunction of the intestinal epithelial barrier.

## 5. Conclusions

**Figure 8:**
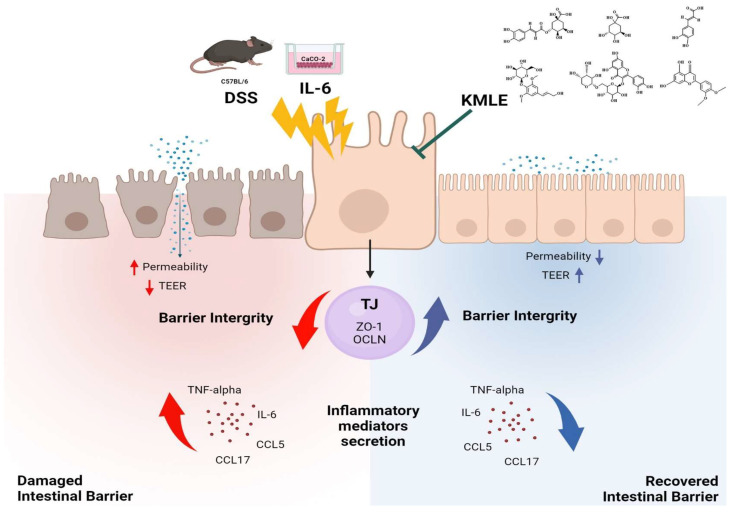
KMLE alleviates barrier dysfunction, improves intestinal barrier function, and reduces inflammation. This figure demonstrates the restoration of the intestinal barrier disrupted by DSS and IL-6 through the therapeutic action of KMLE. DSS and IL-6 compromise barrier integrity by increasing epithelial permeability, reducing TEER, and inducing the release of inflammatory mediators, such as TNF-α, IL-6, CCL5, and CCL17. In contrast, KMLE promotes barrier recovery by upregulating TJPs, including ZO-1 and OCLN, thereby enhancing barrier integrity, suppressing inflammation, and improving TEER. The figure also illustrates the distinct mechanisms of action: yellow arrows represent the damaging effects of DSS and IL-6, leading to increased permeability and inflammation, while green arrows indicate the protective role of KMLE in restoring barrier function. Red arrows show the progression of inflammation and barrier disruption, whereas blue arrows depict the reduction in inflammatory cytokines and the reinforcement of epithelial integrity following KMLE treatment [64].

In conclusion, this study aimed to evaluate the protective effects of KMLE on intestinal mucosal injury in chronic colitis. KMLE was found to restore TJs in the intestinal epithelium, reduce epithelial permeability, and regulate the expression of TJPs. Based on these findings, we suggest that KMLE facilitates the restoration of the impaired intestinal epithelial barrier in chronic colitis by modulating TJ function, providing a potential therapeutic strategy for UC through TJ-targeted interventions (Figure 8).

## Figures and Tables

**Table 1:** DAI Scoring criteria.

Parameter	Weight Loss (%)	Stool Consistency	Rectal Bleeding
0 (Normal)	None	None	None
1	1–5%	Loose stool	Slight (light to dark brown)
2	5–10%	Loose stool	Moderate (dark brown)
3	10–15%	Diarrhea	Severe (diarrhea with red blood)
4	15–20%	No stool output	Gross bleeding (bright red blood throughout the colon)
5	>20%	-	-

**Table 2:** The HPLC-MS/MS data of the chemical components in KMLE.

Peak No.	Rt (min)	Formula	Compound	[M−H]^−^	MS/MS
1	4.45	C_16_H_18_O_9_	Chlorogenic acid	353	191, 179
2	5.89	C_7_H_12_O_6_	Quinic acid	191	173, 155, 111
3	7.55	C_9_H_8_O_4_	Caffeic acid	179	135, 107
4	17.61	C_11_H_14_O_4_	Syringin	317	194, 179, 151
5	38.41	C_26_H_28_O_16_	Quercetin 3-Vicianoside	595	301
6	46.05	C_17_H_14_O_6_	Dimethoxyluteolin	313	298, 255,129

**Table 3:** Molecular docking studies of compounds KMLE with ZO-1 and binding energy.

Binding Ligand	Amino Acid Residue That Interacts	Docking Score (kcal/mol)
Chlorogenic acid	GLY 50, GLU 51, ARG 107, ARG 107, ASP 84, ASN 85	−7.7
Quinic acid	ASN 85, MET 83, ILE 54, VAL 75, SER 53, ARG 107	−5.7
Caffeic acid	ARG 107, ARG 74, ASP 84, ASP 84, ASN 85	−5.8
Syringin	PHE 90, VAL 86, ARG 42	−6.1
Quercetin 3-Vicianoside	ARG 42, ASN 85, ASP 84, ASN 85, ARG 42, PHE 90, VAL 86	−7.9
Dimethoxyluteolin	MET 77, ALA 76, ARG 107, ASP 84, ARG 107, ASP 84, ALA 76, ILE 54, MET 83, VAL 75, SER 53	−7.8

## Data Availability

The original contributions presented in this study are included in the article. Further inquiries can be directed to the corresponding authors.

## Abbreviations

The following abbreviations are used in this manuscript:

IBD	Inflammatory bowel disease
UC	Ulcerative colitis
CD	Crohn’s disease
TJs	Tight junctions
TJPs	Tight junction proteins
ZO	Zonula occludens
JAMs	Junctional adhesion molecules
OCLN	Occludin
5-ASA	5-aminosalicylic acid
SSZ	Sulfasalazine
OSZ	Olsalazine
MSZ	Mesalamine
KML	Korean mistletoe
KMLE	Korean mistletoe extracts
FITC-dextran 4	Fluorescein isothiocyanate-dextran 4kDa
CCK-8	Cell counting kit-8
TNF-α	Tumor necrosis factor-alpha
IL	Interleukin
IFN-γ	Interferon-gamma
DPPH	2,2-diphenyl-1-picrylhydrazyl
ABTS	2,2′-azinobis-(3-ethylbenzothiazoline-6-sulfonate)
AA	Ascorbic acid
TEER	Transepithelial electrical resistance
PBS	Phosphate buffered saline
IF	Immunofluorescence
BSA	Bovine serum albumin
RT-PCR	Reverse transcription-polymerase chain reaction
Ct	Cycle threshold
GAPDH	Glyceraldehyde 3-phosphate dehydrogenase
CCL5	C-C motif chemokine ligand 5
CCL17	C-C motif chemokine ligand 17
DSS	Dextran sodium sulfate
DAI	Disease activity index
H&E	Hematoxylin and eosin
RIPA	Radio-immunoprecipitation assay
SDS-PAGE	Sodium dodecyl sulfate–polyacrylamide gel electrophoresis
HRP	Horseradish peroxidase
RIPA	Enzyme-linked immunosorbent assay
HPLC	High-performance liquid chromatography
LC-MS/MS	Liquid chromatography with tandem mass spectrometry
UPLC	Ultra-performance liquid chromatography
ACN	Acetonitrile
ESI	Electrospray ionization
RCSB PDB	Research Collaboratory for Structural Bioinformatics Protein Data Bank
CSs	Corticosteroids

## References

[1] Xiao Y., Lian H., Zhong X.S., Krishnachaitanya S.S., Cong Y., Dashwood R.H., Savidge T.C., Powell D.W., Liu X., Li Q. (2022). Matrix metalloproteinase 7 contributes to intestinal barrier dysfunction by degrading tight junction protein Claudin-7. Front. Immunol..

[2] Kaplan G.G. (2015). The global burden of IBD: From 2015 to 2025. Nat. Rev. Gastroenterol. Hepatol..

[3] Dowdell A.S., Colgan S.P. (2021). Metabolic Host-Microbiota Interactions in Autophagy and the Pathogenesis of Inflammatory Bowel Disease (IBD). Pharmaceuticals.

[4] Rogler G., Singh A., Kavanaugh A., Rubin D.T. (2021). Extraintestinal Manifestations of Inflammatory Bowel Disease: Current Concepts, Treatment, and Implications for Disease Management. Gastroenterology.

[5] Khaki-Khatibi F., Qujeq D., Kashifard M., Moein S., Maniati M., Vaghari-Tabari M. (2020). Calprotectin in inflammatory bowel disease. Clin. Chim. Acta.

[6] Huang S., Wang X., Xie X., Su Y., Pan Z., Li Y., Liang J., Zhang M., Pan S., Xu B. (2022). Dahuang Mudan decoction repairs intestinal barrier in chronic colitic mice by regulating the function of ILC3. J. Ethnopharmacol..

[7] Krug S.M., Bojarski C., Fromm A., Lee I.M., Dames P., Richter J.F., Turner J.R., Fromm M., Schulzke J.D. (2018). Tricellulin is regulated via interleukin-13-receptor alpha2, affects macromolecule uptake, and is decreased in ulcerative colitis. Mucosal Immunol..

[8] Huang S., Fu Y., Xu B., Liu C., Wang Q., Luo S., Nong F., Wang X., Huang S., Chen J. (2020). Wogonoside alleviates colitis by improving intestinal epithelial barrier function via the MLCK/pMLC2 pathway. Phytomedicine.

[9] Yu M., Wang Q., Ma Y., Li L., Yu K., Zhang Z., Chen G., Li X., Xiao W., Xu P. (2018). Aryl Hydrocarbon Receptor Activation Modulates Intestinal Epithelial Barrier Function by Maintaining Tight Junction Integrity. Int. J. Biol. Sci..

[10] Zihni C., Mills C., Matter K., Balda M.S. (2016). Tight junctions: From simple barriers to multifunctional molecular gates. Nat. Rev. Mol. Cell Biol..

[11] Kuo W.T., Zuo L., Odenwald M.A., Madha S., Singh G., Gurniak C.B., Abraham C., Turner J.R. (2021). The Tight Junction Protein ZO-1 Is Dispensable for Barrier Function but Critical for Effective Mucosal Repair. Gastroenterology.

[12] Zeisel M.B., Dhawan P., Baumert T.F. (2019). Tight junction proteins in gastrointestinal and liver disease. Gut.

[13] Deng J., Zeng L., Lai X., Li J., Liu L., Lin Q., Chen Y. (2018). Metformin protects against intestinal barrier dysfunction via AMPKalpha1-dependent inhibition of JNK signalling activation. J. Cell Mol. Med..

[14] Buckley A., Turner J.R. (2018). Cell Biology of Tight Junction Barrier Regulation and Mucosal Disease. Cold Spring Harb. Perspect. Biol..

[15] Ma L., Ni L., Yang T., Mao P., Huang X., Luo Y., Jiang Z., Hu L., Zhao Y., Fu Z. (2021). Preventive and Therapeutic Spermidine Treatment Attenuates Acute Colitis in Mice. J. Agric. Food Chem..

[16] Huang L., Zheng J., Sun G., Yang H., Sun X., Yao X., Lin A., Liu H. (2022). 5-Aminosalicylic acid ameliorates dextran sulfate sodium-induced colitis in mice by modulating gut microbiota and bile acid metabolism. Cell Mol. Life Sci..

[17] Al-Harbi K.S. (2012). Treatment-resistant depression: Therapeutic trends, challenges, and future directions. Patient Prefer. Adherence.

[18] Szurpnicka A., Zjawiony J.K., Szterk A. (2019). Therapeutic potential of mistletoe in CNS-related neurological disorders and the chemical composition of *Viscum* species. J. Ethnopharmacol..

[19] Ha S.M., Kim J.H., Kim J.W., Kim D.Y., Ha M.S. (2021). The Potential Role of Korean Mistletoe Extract as an Anti-Inflammatory Supplementation. J. Immunol. Res..

[20] Takasawa A., Takasawa K., Murata M., Osanai M., Sawada N. (2023). Emerging roles of transmembrane-type tight junction proteins in cancers. Pathol. Int..

[21] Kienle G.S., Kiene H. (2007). Complementary cancer therapy: A systematic review of prospective clinical trials on anthroposophic mistletoe extracts. Eur. J. Med. Res..

[22] Lee S.H., An H.S., Jung Y.W., Lee E.J., Lee H.Y., Choi E.S., An S.W., Son H., Lee S.J., Kim J.B. (2014). Korean mistletoe (*Viscum album coloratum*) extract extends the lifespan of nematodes and fruit flies. Biogerontology.

[23] Jeong J., Park C.H., Kim I., Kim Y.H., Yoon J.M., Kim K.S., Kim J.B. (2017). Korean mistletoe (*Viscum album coloratum*) extract regulates gene expression related to muscle atrophy and muscle hypertrophy. BMC Complement. Altern. Med..

[24] Fidan I., Ozkan S., Gurbuz I., Yesilyurt E., Erdal B., Yolbakan S., Imir T. (2008). The efficiency of *Viscum album* ssp. album and Hypericum perforatum on human immune cells in vitro. Immunopharmacol. Immunotoxicol..

[25] Jung H.Y., Lee A.N., Song T.J., An H.S., Kim Y.H., Kim K.D., Kim I.B., Kim K.S., Han B.S., Kim C.H. (2012). Korean mistletoe (*Viscum album coloratum*) extract improves endurance capacity in mice by stimulating mitochondrial activity. J. Med. Food.

[26] Hegde P., Maddur M.S., Friboulet A., Bayry J., Kaveri S.V. (2011). *Viscum album* exerts anti-inflammatory effect by selectively inhibiting cytokine-induced expression of cyclooxygenase-2. PLoS ONE.

[27] Cheng Y., Hall T.R., Xu X., Yung I., Souza D., Zheng J., Schiele F., Hoffmann M., Mbow M.L., Garnett J.P. (2022). Targeting uPA-uPAR interaction to improve intestinal epithelial barrier integrity in inflammatory bowel disease. EBioMedicine.

[28] Re R., Pellegrini N., Proteggente A., Pannala A., Yang M., Rice-Evans C. (1999). Antioxidant activity applying an improved ABTS radical cation decolorization assay. Free Radic. Biol. Med..

[29] Cava R., Ladero L. (2024). Using polyphenol-rich extracts from tropical fruit byproducts to control lipid and protein oxidation in cooked chicken models. Eur. Food Res. Technol..

[30] Carini M., Facino R.M., Aldini G., Calloni M., Colombo L. (1998). Characterization of phenolic antioxidants from mate (*Ilex paraguayensis*) by liquid chromatography mass spectrometry and liquid chromatography tandem mass spectrometry. Rapid Commun. Mass. Spectrom..

[31] Bastos K.X., Dias C.N., Nascimento Y.M., da Silva M.S., Langassner S.M., Wessjohann L.A., Tavares J.F. (2017). Identification of Phenolic Compounds from *Hancornia speciosa* (Apocynaceae) Leaves by UHPLC Orbitrap-HRMS. Molecules.

[32] Rivera-Pastrana D.M., Yahia E.M., Gonzalez-Aguilar G.A. (2010). Phenolic and carotenoid profiles of papaya fruit (*Carica papaya* L.) and their contents under low temperature storage. J. Sci. Food Agric..

[33] Sinosaki N.B.M., Tonin A.P.P., Ribeiro M.A.S., Poliseli C.B., Roberto S.B., da Silveira R., Visentainer J.V., Santos O.O., Meurer E.C. (2020). Structural Study of Phenolic Acids by Triple Quadrupole Mass Spectrometry with Electrospray Ionization in Negative Mode and H/D Isotopic Exchange. J. Braz. Chem. Soc..

[34] Sun H., Liu J., Zhang A., Zhang Y., Meng X., Han Y., Zhang Y., Wang X. (2016). Characterization of the multiple components of Acanthopanax Senticosus stem by ultra high performance liquid chromatography with quadrupole time-of-flight tandem mass spectrometry. J. Sep. Sci..

[35] Abu-Reidah I.M., Arraez-Roman D., Lozano-Sanchez J., Segura-Carretero A., Fernandez-Gutierrez A. (2013). Phytochemical characterisation of green beans (*Phaseolus vulgaris* L.) by using high-performance liquid chromatography coupled with time-of-flight mass spectrometry. Phytochem. Anal..

[36] Fathoni A., Saepudin E., Cahyana A.H., Rahayu D.U.C., Haib J. (2017). Identification of nonvolatile compounds in clove (Syzygium aromaticum) from Manado. AIP Conf. Proc..

[37] Lu S., Xu Y., Zhang H., Liu Z., Xu J., Zheng B., Shi D., Qiu F. (2024). Glycyrol Relieves Ulcerative Colitis by Promoting the Fusion of ZO-1 with the Cell Membrane through the Enteric Glial Cells GDNF/RET Pathway. J. Agric. Food Chem..

[38] An J., Liu Y., Wang Y., Fan R., Hu X., Zhang F., Yang J., Chen J. (2022). The Role of Intestinal Mucosal Barrier in Autoimmune Disease: A Potential Target. Front. Immunol..

[39] Yang R., Han X., Uchiyama T., Watkins S.K., Yaguchi A., Delude R.L., Fink M.P. (2003). IL-6 is essential for development of gut barrier dysfunction after hemorrhagic shock and resuscitation in mice. Am. J. Physiol. Gastrointest. Liver Physiol..

[40] Kaminsky L.W., Al-Sadi R., Ma T.Y. (2021). IL-1beta and the Intestinal Epithelial Tight Junction Barrier. Front. Immunol..

[41] Leppkes M., Roulis M., Neurath M.F., Kollias G., Becker C. (2014). Pleiotropic functions of TNF-alpha in the regulation of the intestinal epithelial response to inflammation. Int. Immunol..

[42] Chassaing B., Aitken J.D., Malleshappa M., Vijay-Kumar M. (2014). Dextran sulfate sodium (DSS)-induced colitis in mice. Curr. Protoc. Immunol..

[43] Cai Z.B., Wang S., Li J.N. (2021). Treatment of Inflammatory Bowel Disease: A Comprehensive Review. Front. Med..

[44] Stallmach A., Atreya R., Grunert P.C., Stallhofer J., de Laffolie J., Schmidt C. (2023). Treatment Strategies in Inflammatory Bowel Diseases. Dtsch. Arztebl. Int..

[45] Li Y., Wang K., Li C. (2024). Oxidative Stress in Poultry and the Therapeutic Role of Herbal Medicine in Intestinal Health. Antioxidants.

[46] Ahn J.-H., Shin S.-H., Park S.-M., Choi M.-S., Kim S., Oh J.-H., Yoon S., Park E.-J., Han H.-Y. (2024). Cytotoxicity-reducing and anti-inflammatory effects of a natural herb mixture extract. Mol. Cell. Toxicol..

[47] Zhang M., Li X., Zhang Q., Yang J., Liu G. (2023). Roles of macrophages on ulcerative colitis and colitis-associated colorectal cancer. Front. Immunol..

[48] Kim G.D., Choi J.H., Lim S.M., Jun J.H., Moon J.W., Kim G.J. (2019). Alterations in IL-6/STAT3 Signaling by Korean Mistletoe Lectin Regulate the Self-Renewal Activity of Placenta-Derived Mesenchymal Stem Cells. Nutrients.

[49] Aldinucci D., Colombatti A. (2014). The inflammatory chemokine CCL5 and cancer progression. Mediat. Inflamm..

[50] Panwar S., Sharma S., Tripathi P. (2021). Role of Barrier Integrity and Dysfunctions in Maintaining the Healthy Gut and Their Health Outcomes. Front. Physiol..

[51] Itoh M., Bissell M.J. (2003). The organization of tight junctions in epithelia: Implications for mammary gland biology and breast tumorigenesis. J. Mammary Gland. Biol. Neoplasia.

[52] Umeda K., Ikenouchi J., Katahira-Tayama S., Furuse K., Sasaki H., Nakayama M., Matsui T., Tsukita S., Furuse M., Tsukita S. (2006). ZO-1 and ZO-2 independently determine where claudins are polymerized in tight-junction strand formation. Cell.

[53] Capaldo C.T. (2023). Claudin Barriers on the Brink: How Conflicting Tissue and Cellular Priorities Drive IBD Pathogenesis. Int. J. Mol. Sci..

[54] Gierynska M., Szulc-Dabrowska L., Struzik J., Mielcarska M.B., Gregorczyk-Zboroch K.P. (2022). Integrity of the Intestinal Barrier: The Involvement of Epithelial Cells and Microbiota—A Mutual Relationship. Animals.

[55] Kim K.-Y., Kang Y.-M., Kim T.I., Kim Y.-J., Kim K. (2024). Oncheong-eum alleviated hepatic lipid accumulation and intestinal barrier disruption in nonalcoholic steatohepatitis in vivo model. Mol. Cell. Toxicol..

[56] Kuo W.-T., Zuo L., Turner J. (2021). The Tight Junction Protein Zo-1 Regulates Mitotic Spindle Orientation to Enable Efficient Mucosal Repair. Gastroenterology.

[57] Qian Z.M., Cheng X.J., Wang Q., Huang Q., Jin L.L., Ma Y.F., Xie J.S., Li D.Q. (2023). On-line pre-column FRAP-based antioxidant reaction coupled with HPLC-DAD-TOF/MS for rapid screening of natural antioxidants from different parts of *Polygonum viviparum*. RSC Adv..

[58] Rakoczy K., Kaczor J., Soltyk A., Szymanska N., Stecko J., Sleziak J., Kulbacka J., Baczynska D. (2023). Application of Luteolin in Neoplasms and Nonneoplastic Diseases. Int. J. Mol. Sci..

[59] Huang J., Xie M., He L., Song X., Cao T. (2023). Chlorogenic acid: A review on its mechanisms of anti-inflammation, disease treatment, and related delivery systems. Front. Pharmacol..

[60] Wang W., Sun W., Jin L. (2017). Caffeic acid alleviates inflammatory response in rheumatoid arthritis fibroblast-like synoviocytes by inhibiting phosphorylation of IkappaB kinase alpha/beta and IkappaBalpha. Int. Immunopharmacol..

[61] Kim Y.W., Yu S.N., Kim K.Y., Kim S.H., Park B.B., Oh H.C., Kim D.S., Park K.I., Ahn S.C. (2023). Biological characterization of mulberry leaves bioconverted with *Viscozyme* L. Mol. Cell. Toxicol..

[62] Hossin A.Y., Inafuku M., Takara K., Nugara R.N., Oku H. (2021). Syringin: A Phenylpropanoid Glycoside Compound in Cirsium brevicaule A. GRAY Root Modulates Adipogenesis. Molecules.

[63] Ercan L., Doğru M. (2022). Antioxidant and Antimicrobial Capacity of Quinic Acid. Bitlis Eren Üniversitesi Fen Bilim. Derg..

[64] Kim W. (2025). KMLE Restores Intestinal Barrier Integrity and Reduces Inflammation. https://BioRender.com/s27m519.

